# Anogenital Distance Plasticity in Adulthood: Implications for Its Use as a Biomarker of Fetal Androgen Action

**DOI:** 10.1210/en.2014-1534

**Published:** 2014-11-06

**Authors:** Rod T. Mitchell, Will Mungall, Chris McKinnell, Richard M. Sharpe, Lyndsey Cruickshanks, Laura Milne, Lee B. Smith

**Affiliations:** MRC Centre for Reproductive Health, University of Edinburgh, The Queen's Medical Research Institute, Edinburgh, EH16 4TJ, United Kingdom

## Abstract

Androgen action during the fetal masculinization programming window (MPW) determines the maximum potential for growth of androgen-dependent organs (eg, seminal vesicles, prostate, penis, and perineum) and is reflected in anogenital distance (AGD). As such, determining AGD in postnatal life has potential as a lifelong easily accessible biomarker of overall androgen action during the MPW. However, whether the perineum remains androgen responsive in adulthood and thus responds plastically to perturbed androgen drive remains unexplored. To determine this, we treated adult male rats with either the antiandrogen flutamide or the estrogen diethylstilbestrol (DES) for 5 weeks, followed by a 4-week washout period of no treatment. We determined AGD and its correlate anogenital index (AGI) (AGD relative to body weight) at weekly intervals across this period and compared these with normal adult rats (male and female), castrated male rats, and appropriate vehicle controls. These data showed that, in addition to reducing circulating testosterone and seminal vesicle weight, castration significantly reduced AGD (by ∼17%), demonstrating that there is a degree of plasticity in AGD in adulthood. Flutamide treatment increased circulating testosterone yet also reduced seminal vesicle weight due to local antagonism of androgen receptor. Despite this suppression, surprisingly, flutamide treatment had no effect on AGD at any time point. In contrast, although DES treatment suppressed circulating testosterone and reduced seminal vesicle weight, it also induced a significant reduction in AGD (by ∼11%), which returned to normal 1 week after cessation of DES treatment. We conclude that AGD in adult rats exhibits a degree of plasticity, which may be mediated by modulation of local androgen/estrogen action. The implications of these findings regarding the use of AGD as a lifelong clinical biomarker of fetal androgen action are discussed.

The most common male reproductive disorders that manifest at birth (cryptorchidism and hypospadias) or in adulthood (low sperm counts and testicular germ cell cancer) may be increasing in prevalence and may have a common origin in fetal life (1). Establishing whether fetal events have negatively affected the reproductive health of men is challenging because it requires a means of “seeing back in time” (2); until recently this has proven impossible. However, it has been long established in rodents that anogenital distance (AGD) (the measured distance between the anus and the genitals) reflects fetal androgen action (3); AGD in males is approximately double that of females. What has generated renewed interest in AGD has been the discovery that it reflects fetal androgen exposure only within a discrete “masculinization programming window” (MPW), which also determines the adult size of the testis, prostate, seminal vesicles, and penis (4–7). Moreover, disorders such as hypospadias and cryptorchidism are also linked to reduced androgen action during the same MPW, such that AGD in adulthood could provide a snapshot of androgen action during this critical period in fetal life (reviewed in Ref. 8).

AGD has several attractive properties in terms of its potential clinical use, because it is easily accessible and noninvasive. The rodent studies on AGD and MPW outlined above have prompted several clinical studies of whether AGD is associated with similar reproductive phenotypes in boys/men; these have identified associations between reduced AGD and hypospadias (9, 10), cryptorchidism (10–13), and penis size in both boys (10, 13, 14) and men (15, 16), as well as with lower sperm counts/infertility in normal young men and andrology clinic patients (17, 18). The association between AGD and several clinical manifestations has meant that use of AGD as a clinically useful biomarker has gained some traction, although many questions remain unanswered. Data are only just emerging regarding the natural variation in AGD across the normal population (14), and this is restricted to early postnatal life. If AGD is to be used in patients as a reliable biomarker of fetal androgen action in the MPW, more understanding about how AGD might be (secondarily) influenced during postnatal life through to adulthood is needed.

Whereas the initial studies on rodents described the utility of AGD as a lifelong indicator of androgen action during fetal development and specifically in the MPW, they also established that this only held true if the androgen-dependent postnatal growth potential was maximized (4, 5, 19). Failure to expose the perineum (AGD) and reproductive organs to normal concentrations of androgens in postnatal life reduced the final size of these tissues (20–22). These data suggest that there might be a degree of plasticity in adult AGD, at least if ambient androgen levels are subnormal, such as in hypogonadism. This possibility has not previously been tested in longitudinal studies in either rodents or humans, nor has the possibility that alterations of the androgen-estrogen balance might affect the size of the already established AGD.

To address this, we have determined whether AGD demonstrates bidirectional plasticity in adulthood in rats by treatment with either the antiandrogen flutamide or the potent estrogen diethylstilbestrol (DES). Our findings demonstrate that significant hormone-dependent reductions in AGD are achievable in adulthood, which has implications for the potential use of AGD as a lifelong clinical biomarker of fetal androgen action.

## Materials and Methods

### Ethics statement

Rats were housed and bred under standard conditions of care. Experiments were conducted, after local ethical approval, under UK Home Office license number PPL 60/4200.

### Treatments

#### Castration

Rats were anesthetized by inhalation of isoflurane and castrated using a scrotal incision. Analgesia consisted of buprenorphine (45 μg/kg) at the time of surgery followed by carprofen (1 ml/L) in the drinking water for 24 hours postsurgery.

#### DES

Rats were treated by subcutaneous injection, every 3 days with a final concentration of 100 μg/kg DES (Sigma-Aldrich) in 0.001% ethanol in corn oil (Sigma-Aldrich) or vehicle.

#### Flutamide

Rats were treated by daily oral gavage with a final concentration of 100 mg/kg/d flutamide (Sigma-Aldrich) in 2.5% dimethylsulfoxide (Sigma-Aldrich) in corn oil; or vehicle.

### Experimental design

To determine AGD plasticity, 84 adult Wistar rats (6 groups of n = 12 males/group; 1 group of n = 12 randomly cycling females) (all 9 weeks of age) were separated into 1 of 3 cohorts: (1) intact male, castrated male, and female; (2) DES-exposed and vehicle (for DES)-exposed; and (3) flutamide-exposed and vehicle (for flutamide)-exposed. AGD was measured as the distance between the base of the phallus and the anterior margin of the anus using digital calipers (Faithfull Tools). To prevent bias, all males within each cohort were randomly allocated to treatment groups, and the individuals measuring AGD were blinded to treatments. Body weight and AGD of each individual were determined weekly for a period of 9 weeks, beginning 1 week after commencement of treatment. Those groups receiving treatments were treated for 5 weeks, followed by a 4-week washout period. Three individuals from each group were culled at the end of week 5 to confirm that treatments were successful. The remaining 9 individuals were culled at the end of week 9.

### Measurements of Testosterone and LH

Sera were separated and stored at −20°C. Serum testosterone was determined using an RIA as described previously (23); the average intra-assay coefficient of variation was 6.1% with a limit of detection of 45 pg/mL. The antibody has very low cross-reactivity with other hormones (dihydrotestosterone, 1%; nortestosterone, <1%; SHBG, 0.5%; and progesterone and other steroids, <0.01%). LH was determined as described previously (24). In brief, the LH assay was a sandwich ELISA using an anti-LH β-chain monoclonal antibody (code 518B7; J.F. Roser, Department of Animal Science, University of California, Davis, Davis, CA) as the capture antibody. The signal antibody was biotinylated anti–β-chain LH monoclonal antibody (code 5303; Medix Biochemica). The lower limit of detection was 50 pg/mL, and the cross-reactivity was <0.01% against mouse FSH and TSH. The Between-batch percent coefficient of variation was <10%.

### Statistical analysis and figures

Data were analyzed using one-way or two-way ANOVA as appropriate using GraphPad Prism (version 6; GraphPad Software Inc). Values are expressed as means ± SEM. Figures were compiled using Adobe Illustrator CS6 (Adobe System Inc.).

## Results

**Figure 1.**
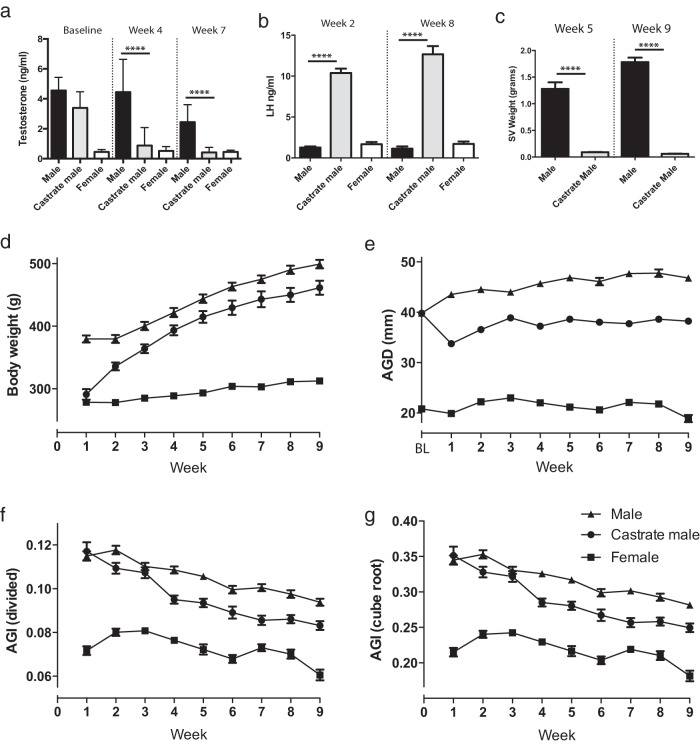
Castration reduces AGD in the adult rat. A, Circulating testosterone from male, castrated male, and female rats assayed at baseline, week 4, and week 7 of the study. B, Circulating LH from male, castrated male, and female rats assayed at weeks 2 and 8 of the study. C, Seminal vesicle (SV) weights of males vs castrated males at week 5 and week 9 of the study. D, Body weight of the 3 groups across the 9 weeks of the study. E, AGD of the 3 groups across the 9 weeks of the study. BL, baseline. F, AGI (calculated by dividing AGD by body weight) of the 3 groups across the 9 weeks of the study. G, AGI (calculated by dividing AGD by the cube root of body weight) of the 3 groups across the 9 weeks of the study. A, B, and C, one-way ANOVA; D, E, F, and G, two-way ANOVA. ****, *P* < .0001.

To determine whether AGD exhibits plasticity in adulthood and whether sex hormones play a significant role in this, a cohort of adult male rats were castrated and compared with intact control adult males and intact control adult females over a 9-week period. Circulating testosterone was significantly reduced when measured 4 weeks and 7 weeks after castration, falling to levels equivalent to those of control females (Figure 1A). Consistent with this finding, castration resulted in a significant increase in circulating LH when assayed at weeks 2 and 8 (Figure 1B) and a significant reduction in seminal vesicle weight (a biomarker of systemic androgen action) when determined at weeks 5 and 9 (Figure 1C).

Although male rats entered the study when reproductively mature, a steady increase in body weight was observed in control males throughout the 9-week period of study (Figure 1D), consistent with the published literature (25). One week after castration, the body weight of castrated males had fallen to female values (possibly as a response to undergoing surgery, in addition to loss of the androgen drive) but thereafter increased steadily to control male values at week 3 after castration, which were maintained for the remainder of the study (Figure 1D).

### AGD is reduced after castration of adult male rats

Control males demonstrated a small but progressive increase in AGD throughout the 10-week period of study, whereas, in contrast, females showed no increase in AGD throughout the experiment (Figure 1E). Castrated males showed a significant reduction (∼17%) in AGD compared with that of control males at all time points examined. However, the AGD of castrated males never reduced to a size close to that of AGD in females (Figure 1E). To allow for changes in body weight, we calculated the anogenital index (AGI) using the 2 well-established methods: (1) dividing AGD by body weight and (2) dividing AGD by the cube root of body weight. Both measures gave broadly similar results (Figure 1, F and G). Castrated males showed a significant difference in AGI compared with control males. However, this was generated as a composite value, reflecting changes in both body weight and AGD. The findings in castrated males suggest that, in such extreme circumstances, adult AGD does exhibit a degree of plasticity.

### AGD plasticity in adulthood is unresponsive to suppression of androgen signaling

**Figure 2.**
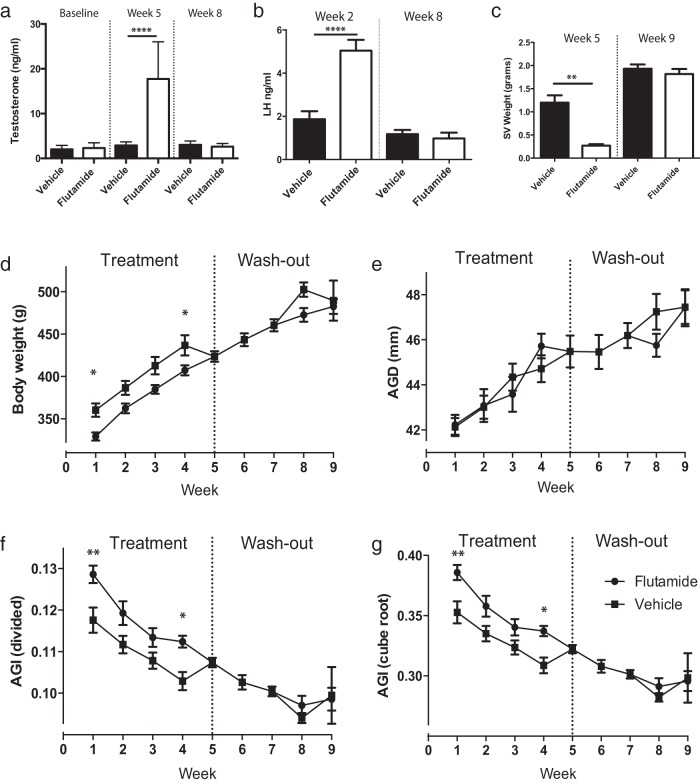
Blocking androgen action via AR does not affect AGD in the adult male rat. A, Circulating testosterone from flutamide-treated male rats vs vehicle-treated controls assayed at baseline, week 5, and week 8 of the study. B, Circulating LH from flutamide-treated male rats vs vehicle-treated controls assayed at weeks 2 and 8 of the study. C, Seminal vesicle (SV) weights of flutamide-treated male rats vs vehicle-treated controls at week 5 and week 9 of the study. D, Body weight of flutamide-treated male rats vs vehicle-treated controls across the 9 weeks of the study. E, AGD of flutamide-treated male rats vs vehicle-treated controls across the 9 weeks of the study. F, AGI (calculated by dividing AGD by body weight) of flutamide-treated male rats vs vehicle-treated controls across the 9 weeks of the study. G, AGI (calculated by dividing AGD by the cube root of body weight) of flutamide-treated male rats vs vehicle-treated controls across the 9 weeks of the study. A, B, and C, one-way ANOVA; D, E, F, and G, two-way ANOVA. *, *P* < .05; **, *P* < .01; ****, *P* < .0001.

To determine the mechanism underpinning adult AGD plasticity, a cohort of intact adult male rats was treated with the potent androgen receptor (AR) antagonist flutamide for 5 weeks, followed by a 4-week washout period of no treatment. These animals were compared with a similar cohort treated with vehicle. Flutamide treatment significantly increased circulating testosterone levels compared with those of controls when measured at week 5 (Figure 2A), because of inhibition of negative feedback at the level of the hypothalamus/pituitary, reflected in a significant increase in circulating LH (Figure 2B); both LH and testosterone returned to normal levels at week 8 (during the washout period) (Figure 2, A and B). Despite the flutamide-triggered increase in circulating testosterone, seminal vesicle weight was significantly reduced at 5 weeks, demonstrating that the flutamide had successfully functioned as an AR antagonist in peripheral tissues (Figure 2C). Seminal vesicle weight had returned to normal by week 9, demonstrating that the washout period successfully relieved antagonism of AR (Figure 2C).

Male rats treated with vehicle showed a profile of increasing body weight similar to that of intact control animals (Figure 2D). In contrast, although body weight increased in flutamide-treated males, it was significantly reduced compared with that of vehicle controls at weeks 1 and 4 of treatment (Figure 2D), with full recovery from weeks 5 to 9 (washout). Despite perturbation of AR signaling in peripheral tissues, AGD did not differ between flutamide-treated males and controls at any time point (Figure 2E). Consequently, calculation of AGI, which did show a significant difference between vehicle- and flutamide-treated animals at weeks 1 and 4 of treatment, was entirely the result of changes in body weight (Figure 2, F and G).

### AGD plasticity in adulthood is affected by estrogens

**Figure 3.**
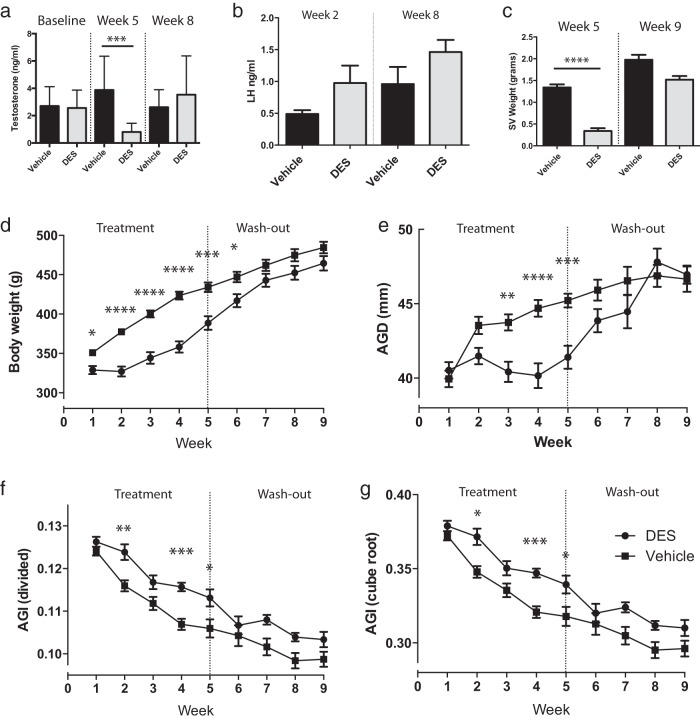
Increasing estrogen signaling reduces AGD in the adult male rat. A, Circulating testosterone from DES-treated male rats vs vehicle-treated controls assayed at baseline, week 5, and week 8 of the study. B, Circulating LH from DES-treated male rats vs vehicle-treated controls from weeks 2 and 8 of the study. C, Seminal vesicle (SV) weights of DES-treated male rats vs vehicle-treated controls at week 5 and week 9 of the study. D, Body weight of DES-treated male rats vs vehicle-treated controls across the 9 weeks of the study. E, AGD of DES-treated male rats vs vehicle-treated controls across the 9 weeks of the study. F, AGI (calculated by dividing AGD by body weight) of DES-treated male rats vs vehicle-treated controls across the 9 weeks of the study. G, AGI (calculated by dividing AGD by the cube root of body weight) of DES-treated male rats vs vehicle-treated controls across the 9 weeks of the study. A, B, and C, one-way ANOVA; D, E, F, and G, two-way ANOVA. *, *P* < .05; **, *P* < .01; ***, *P* < .001; ****, *P* < .0001.

Because AGD plasticity was not mediated by a reduction in androgen action, we next examined whether excess estrogen signaling could modulate AGD in adulthood. A separate cohort of male rats was treated with DES for 5 weeks, followed by a 4-week washout period of no treatment. These animals were compared to a similar cohort treated with vehicle. DES treatment significantly reduced circulating testosterone concentrations when measured at week 5 of treatment, but levels had returned to normal when measured at week 8 (washout) (Figure 3A). Circulating LH was unchanged at both week 2 (although there was a trend toward increased LH (*P* = .0849) and week 8 (Figure 3B). Consistent with the DES-induced reduction in testosterone levels, seminal vesicle weight was also significantly reduced at week 5, but also returned to normal by week 9 in the washout period (Figure 3C).

Male rats treated with vehicle (for DES) showed a profile of increasing body weight comparable to that of intact control animals (Figure 3D). In contrast, although body weight also increased in DES-treated males, it was reduced significantly compared with that of vehicle controls at weeks 1 to 6 of treatment, but was similar to that of controls by week 7 (Figure 3D).

Male rats treated with DES showed a significant reduction in AGD at weeks 3, 4, and 5 of treatment (Figure 3E). Cessation of treatment resulted in a return to control AGD size 1 week later, which was maintained for the remainder of the study (Figure 3E). AGI was significantly different between DES-treated and vehicle control animals, demonstrating that although DES affected both body weight and AGD, the correlation between body weight and AGD was not linear (Figure 3, F and G).

## Discussion

Androgen exposure during the MPW determines the maximum “potential” adult size to which androgen-dependent reproductive organs and AGD can grow (8). However, androgens are also important drivers of this growth (4, 5, 19), and although previous studies have implied that AGD could exhibit plasticity postnatally (20–22), the concept of AGD plasticity in adulthood has not previously been explored directly. In the first longitudinal study of its type, we now show that AGD exhibits a degree of plasticity, even in adulthood.

Castration of male rats led to a significant reduction in AGD 1 week later. This provided the first evidence that AGD could also be actively reduced in adulthood by changes in steroid hormone action. We initially presumed that the castration effect was the result of androgen withdrawal, but when we treated intact adult rats with the AR antagonist flutamide, we found no such effect on AGD, despite confirming (via seminal vesicle weight reduction and increased circulating LH (indicative of inhibition of androgen/AR feedback in the brain) that peripheral androgen action had been considerably attenuated. However, 1 difference between the 2 approaches (castration vs AR suppression) is in the intactness of the hypothalamic-pituitary-gonadal axis. Thus, although LH levels were significantly increased after flutamide treatment, levels did not climb as high as LH levels after castration. This difference is probably explained by the intact compensatory homeostatic mechanisms in flutamide-exposed males (flutamide treatment induces a compensatory increase in testosterone production, which would itself attenuate LH production in the flutamide-treated rats). Together these data suggest that flutamide-mediated suppression of androgen signaling was not as complete as that after castration, raising the possibility that the lack of impact on AGD simply reflects a failure to sufficiently suppress androgen action.

Whether this lack of effect on AGD is mechanistic (the perineum is unresponsive to androgen suppression per se) or dosage-related (the perineum is unresponsive to androgen suppression at this dose) requires further investigation, but whichever explanation is correct, the lack of impact of flutamide on AGD at the high doses used in this study supports our proposition that suppression of androgen action via AR is not a *primary* mechanism underpinning AGD plasticity in adulthood.

In support of this proposition, we note that DES treatment also reduces circulating testosterone levels (consistent with the published literature [26]) and thus attenuates androgen signaling. With the use of seminal vesicle weight as an accepted biomarker of peripheral androgen action, the level of suppression of androgen signaling (revealed by similar seminal vesicle weights) was comparable in flutamide- and DES-treated rats (but not as complete as that after castration). Despite this similarity, a reduction in AGD (∼11%) was *only* found in the DES-treated animals, suggesting that a relative increase in estrogen signaling is the primary mechanism underpinning AGD plasticity in adulthood.

There are 2 possible explanations for the DES-induced reduction in AGD: first, that excess estrogen signaling per se explains the DES-induced reduction in AGD; or second, that it results from alteration of the normal androgen-estrogen balance. Because increased estrogen signaling cannot account for the castration-induced decrease in AGD, we propose that a high androgen-low estrogen profile may be necessary to maintain/achieve maximal-sized AGD. Put another way, the presence of abundant androgens is sufficient to suppress aberrant estrogen signaling, and suppression of estrogen signaling is the key factor in ensuring that AGD is maximized; by inference, failure to sufficiently oppose estrogen signaling leads to a reduction in AGD. Whether this is indeed the case, and whether this also applies to other androgen-responsive reproductive organs requires significant further investigation. Our findings with DES raise the possibility that other compounds with weaker estrogenic activity might be similarly capable of altering AGD in adult male rats. This possibility remains to be explored, but we consider it unlikely because weakly estrogenic compounds, such as alkylphenolic or bisphenolic compounds, do not cause suppression of either endogenous testosterone or of AR expression even at high (milligram) doses compared with the potent estrogen DES (this study and Refs. 27, 28). In addition, our study did not explore whether treatment with androgens could alter AGD in adulthood. However, in a pilot short-term (10-day) study involving high-dose testosterone treatment, we found no significant change in AGD despite a profound increase in seminal vesicle weight (unpublished data, R.M.S., L.B.S., R.T.M.), so we consider it unlikely that similar treatment for a longer period of 5 weeks, as in the present studies, would induce any major change in AGD.

Although this study used high doses of flutamide and DES to investigate AGD plasticity in a rat model, as a proof of concept study, these data support several important and novel observations. (1) AGD is not irrevocably fixed by adulthood, but changes to a small but progressive extent throughout adulthood in rats dependent upon age. (2) Consequently, comparison of AGD between rats of different adult ages could result in misleading conclusions, an important observation for future analysis of endocrine disruptors in the rat. (3) AGD exhibits a small degree (11%–17%) of plasticity in adulthood and can be altered by sex steroid manipulations, at least when these are extreme. (4) Both flutamide and DES perturb normal gain of body weight, which acts as a confounder for the utility of AGI. (5) The correlation between body weight and AGD (AGI) is not linear, even in untreated animals, and, therefore, AGI determination in rats may be a poor compensator for differences in body weight. (6) The current presumption that AGD provides a lifelong readout of androgen exposure during the MPW in rats may not be tenable in situations in which major/extreme alterations in circulating blood androgens and/or estrogens occur.

In conclusion, although this study used extreme manipulations of sex steroid exposure in adult rats to induce AGD changes, in a clinical setting it may be prudent to consider that, when changes in androgen-estrogen balance have occurred (eg, obesity, aging, and late-onset hypogonadism), secondary changes in AGD may also have occurred. This concept necessitates further investigation in human cohorts.
